# Knowledge and practice of community pharmacists towards SGLT2 inhibitors

**DOI:** 10.12688/f1000research.122170.1

**Published:** 2022-06-15

**Authors:** Abdelrahim Alqudah, Muna Oqal, Ahmad Al-Samdi, Esam Qnais, Mohammed Wedyan, Majd Abu Gneam, Roaa Alnajjar, Manar Alajarmeh, Elaf Yousef, Omar Gammoh

**Affiliations:** 1 Department of clinical pharmacy and pharmacy practice, Faculty of pharmaceutical sciences, Hashemite University, Zarqa, Zarqa, P.O box 330127, Zarqa 13133, Jordan, Jordan; 2 Department of Pharmaceutics and Pharmaceutical Technology, Faculty of Pharmaceutical sciences, Hashemite University, Zarqa, Zarqa, 13133, Jordan; 3 Department of Adult Health Nursing, Princess Salma Faculty of Nursing, Al al-Bayt University, Al-Mafraq, Zarqa, 13133, Jordan; 4 Department of Biology and Biotechnology, Faculty of Science, Hashemite University, Zarqa, Zarqa, 13133, Jordan; 5 Department of Clinical Pharmacy and Pharmacy Practice, Yarmouk University, Irbid, Zarqa, 13133, Jordan

**Keywords:** Diabetes, SGLT2 inhibitors, Pharmacists, Jordan

## Abstract

**Background: **Sodium/glucose cotransporter 2 (SGLT2) inhibitors are a new class of oral anti-diabetic drugs which improve glycaemic control in type 2 diabetes mellitus (T2DM) by preventing the kidney from reabsorbing glucose back to blood. Community pharmacists have long-term relationships with most of their chronic patients, so they play a key role in care for people with diabetes. Therefore, the objective of this study was to assess pharmacists’ knowledge and practice towards SGLT2 inhibitors.

**Methods**: A cross-sectional study was conducted to meet the study objectives. A convenience sample of 348 community pharmacists in Jordan was recruited. knowledge and practice were assessed using a self-administered questionnaire created for the purpose of this study.

**Results**: A total of 400 community pharmacists were reached, of whom 348 answered the survey (response rate 87%). The results indicated that SGLT2 inhibitors knowledge score among community pharmacists in Jordan was 6.61 (out of 12). Factors like age, gender, location of the pharmacy, years of pharmacists’ experience had no effect on knowledge score; however, pharmacists who attended training courses on diabetes had higher knowledge scores. Additionally, pharmacists’ dispensing practice toward SGLT2 inhibitors had insufficient knowledge, such as lack of knowledge about the superiority of SGLT2 inhibitors over other anti-diabetics and inability to give the best advice to patients.

**Conclusions**: Our findings reflect a moderate knowledge among community pharmacists about SGLT2 inhibitors which may negatively affect the patients’ outcome; thus, continuous education for the pharmacists is essential.

## Introduction

Type 2 diabetes mellitus (T2DM) is a metabolic disorder affecting carbohydrate, fat, and protein metabolism. ^1^ This condition results from inadequate insulin secretion, insulin resistance, or both, often at later stages in life. ^2^^,^^3^ T2DM is characterised by chronic hyperglycaemia and dyslipidaemia which results in the development of renal disease, cardiovascular disease and microvascular complications. ^4^ T2DM management should begin in most patients with lifestyle changes which is often followed up by metformin monotherapy. If target blood glucose is not achieved within 3 months, another hypoglycaemic agents can be added. ^5^

The expression of sodium-glucose cotransporter-2 (SGLT2) proteins selectively occurs in the kidney specifically in the proximal convoluted tubule. Roughly 90% of the absorption of the filtered glucose is under the responsibility of these transporters. Therefore, SGLT2 represents an ideal target and effective options for the treatment of diabetes. ^6^^,^^7^ In patients with T2DM, the renal threshold for reabsorption of glucose is increased above 180 mg/dL (serum glucose concentration), which corresponds to the normal renal threshold reabsorption level of glucose. In addition, it has been reported that the expression of these transporters could be up-regulated in T2DM patients which has the potential to cause a maladaptive response that in turn deteriorates hyperglycaemia. ^8^ Accordingly, the selective SGLT2 inhibitors can cause a reduction to the respective threshold to as low as 40 to 120 mg/dL. ^9^ In fact, the combination of metformin and a SGLT2 inhibitor may be beneficial for patients who are at a high risk of experiencing hypogylcemia, because the hypoglycaemic effect of SGLT2 inhibitors is small in comparison to insulin and sulfonylureas. ^10^ Currently, the Food and Drug Administration (FDA) has approved three SGLT2 selective inhibitors for use in mono, dual, and triple therapy: Canagliflozin, Dapagliflozin and Empagliflozin, and they recently were introduced to the Jordan market. ^7^^,^^11^

The prevalence of T2DM is high in the Middle East because of the development, diet patterns, and the rapid expansion in economy. ^12^ Jordan has a high prevalence of T2DM and has been higher than the global average, as the prevalence rate for diabetes among Jordanian population aged between 25 and 70, was increased from 17.1% in 2004 to 23.7% in 2017. ^13^ Therefore, one treatment approach for improving glycaemic control in T2DM is enhancing the quality of pharmaceutical interventions, increasing patients’ compliance, and using the most recently approved anti-diabetic classes SGLT2 inhibitors. ^14^^,^^15^ In fact, community pharmacists have long-term relationships with most of their chronic patients, because patients obtain their chronic medications and diabetes supplies from community pharmacies; therefore, they play a key role in the care of people with diabetes. A large body of evidence revealed that community pharmacists education about diabetes and its medications has improved patients’ glucose and lipid profile in addition to improving cardiovascular outcomes and other complications. ^16^^–^^18^ Thus, the lack of professional pharmaceutical knowledge might have a negative impact on patients with diabetes’ outcomes. Accordingly, assessing the current related basic pharmacological knowledge and dispensing practice of community pharmacists of SLGT2 inhibitors, as well as uncovering the weakest areas related to this new anti-diabetic class, has the potential to improve the pharmaceutical outcomes of these medications. Therefore, the objectives of this study were to assess pharmacists’ knowledge and dispensing practice toward SGLT2 inhibitors and explore their perception and dispensing practice.

## Methods

### Design and data collection

A cross-sectional design was conducted to meet the study objectives. Data collection was performed between March and September 2020 using a self-administrated questionnaire. The participant pharmacists in our study were visited by the researchers to establish a relationship with them and explain the goals of this research prior to study commencement. Participants were recruited from community pharmacies located among different cities in Jordan including: Amman, Zarqa, Ajloun, Irbid, and Salt, and countryside and city were both targeted based on socio-economic status among each city. The questionnaire was distributed face-to-face with participants at their workplace by four male and female pharmacy students well-trained on data collection and study methods from the Hashemite University. A signed informed consent was obtained from the participants as a pre-requisite to proceed with participation. Participants were interviewed alone for 20-30 minutes to answer the survey questions without anybody beside them and no audio or video recordings were used.

### Sample size

The sample size was calculated based on a 95% confidence level, and 5% confidence interval. The total community pharmacies pool in Jordan is 2,864; the sample size calculation revealed the need for at least 346 community pharmacies pharmacists.

### Ethical considerations

The study was approved by the Institutional Review Board at the Hashemite University in Jordan (Reference number: 8/5/2019/2020). Participants’ involvement in the study was voluntary, and they were informed that they had the right to withdraw from the study at any time.

### Development of the survey questionnaire

A self-administered questionnaire was created especially for the purpose of this study, and was validated by a group of experts constituted of two pharmacologists, one endocrinologist, and two pharmacists. This questionnaire was composed of 28 questions which were divided into three sections. The first section consisted of eight questions about demographics data including gender, age, pharmacy location, working time, years of experience, holding a postgraduate degree, attending training courses on diabetes, and the bachelor degree (BSc) of the pharmacists; BSc in Pharmacy or BSc in Pharmacy doctor (Pharm D). The second section was composed of 12 questions regarding the knowledge of community pharmacists about SGLT2 inhibitors as shown in Table 2. The third section was composed of nine question in relation to practice of community pharmacists toward SGLT2 inhibitors including their impression about this group of medications, the frequency at which they received prescriptions for these agents, the best advice for the patients while using these agents, the obstacles they faced while dispensing these agents, how they evaluated their knowledge about SGLT2 inhibitors, how they assessed their need for training courses about SGLT2 inhibitors, how they assessed their need to attend training courses about SGLT2 inhibitors, and what was the source of information they used to improve their knowledge about SGLT2 inhibitors. The questionnaire was pretested for reliability through the pilot study. The views scale was calculated and showed an excellent reliability with a Cronbach’s alpha of 0.885. Piloting of the questionnaire was performed to assess the comprehension and accuracy of the questions in relation to the research topic, identify possible redundancy among the 28 questions, and ensure the usability of the data collection method.

### Data analysis

Data were coded and incorporated into Statistical Package for Social Sciences (SPSS) version 24.0 (SPSS Inc., Chicago, IL, USA) software after extracting it from Google forms. Demographics numerical variables were described using mean and standard deviation. Knowledge difference in demographics with two categories was tested using independent t-tests; however, knowledge difference in demographics with more than two categories was examined using a one-way analysis of variance (ANOVA) followed by a Scheffe posthoc test. Significance was considered when p<0.05.

### Knowledge scale validity and score

The knowledge scale was assessed for validity by examining the content validity index (CVI). Five experts were consulted for their opinion for each scale item including relevancy, clarity, and simplicity and scores from 1 (not all) to 4 (strongly agreed). Questions were tested/re-tested for their clarity and simplicity by interviewing 20 participants. The CVI were ranged from 0.8-0.9 and it was deemed that the scale was valid. SGLT2 inhibitors knowledge score was 6.61 (SD=2.22, range 1-12).

## Results

### Demographics

A total of 400 community pharmacists were reached (response rate 87%), and 348 completed this survey. Most of the participant pharmacists were less than 30 years old (n=225, 64.7%). Female participants were slightly predominant (n=203, 58.3%) compared to male participants (n=145, 41.7%). The vast majority of the participants were working in city areas (n=331, 95.1%), their working experience was 0-4 years (n=191, 54.9%), and had no postgraduate degree (n=304, 87.4%). Most participants had not attended training courses on diabetes (n=255, 73.3%) and they had a bachelor’s degree in pharmacy (n=307, 88.2%). More details about the sociodemographic characteristics are presented in Table 1.

**Table 1.** demographical details, n=348.

Factor	Categories	Total No. (%)
Age	Up to 29	225 (64.7)
	30 or more	123 (35.3)
Gender	Female	203 (58.3)
	Male	145 (41.7)
Pharmacy location	City	331 (95.1)
	Countryside	17 (4.9)
Working time	Morning	177 (50.9)
	Afternoon or night	171 (49.1)
Years of experience	0-4 years	191 (54.9)
	5-9 years	85 (24.4)
	10 years or more	72 (20.7)
Postgraduate degree	Higher diploma or master	44 (12.6)
	Have no postgraduate degree	304 (87.4)
Training courses in diabetes	Yes	93 (26.7)
	No	255 (73.3)
Bachelor’s degree major	Pharmacy	307 (88.2)
	Pharm D	41 (11.8)

### Pharmacists’ knowledge level about SGLT2

Community pharmacists’ knowledge score was 6.61 (SD=2.22, range 1-12) which is around the knowledge scale average. The results of this study showed that only 32.5% of the pharmacists provided an adequate answer that SGLT2 inhibitors decrease blood pressure. In addition, 34.5% of the pharmacists knew the best consultation to the patients using SGLT2 inhibitors is to keep genital area clean to avoid infection. Moreover, only 38.5% of them knew that patients with diabetes and hypertension are the best candidates for SGLT2 inhibitor agents. On the other hand, pharmacists provided a good knowledge that SGLT inhibitors are contraindicated in patients with renal failure, and they have a better protective effect in patients with diabetes and hypertension compared to sulfonylurea (63.5% and 63.8%; respectively). The highest knowledge of the pharmacists was for their response to the question related to the dosage form of SGLT2 inhibitors, which is tablets (92.5%). Further details about the pharmacists’ knowledge are provided in Table 2.

**Table 2.** Community pharmacists’ knowledge about SGLT2 inhibitors.

Question	Adequate answer	Number of adequate answers n (%)
What is the effect of SGLT2 inhibitors on blood pressure?	Decrease blood pressure	113 (32.5)
What is the best advice you can give to the patients who are using SGLT2 inhibitors?	Keep genital area clean to avoid infection	120 (34.5)
To which patients SGLT2 inhibitors group is prescribed the best?	Patients with diabetes and hypertension	134 (38.5)
How do SGLT2 inhibitors work?	Increase glucose renal excretion	143 (41.1)
SGLT2 stands for?	Sodium glucose transporter protein	149 (42.8)
What is the most common side effect of SGLT2 inhibitors?	Urinary tract infection	153 (44)
What is the effect of SGLT2 inhibitors on body weight?	Decrease body weight	175 (50.3)
When SGLT2 inhibitors group is used	After metformin	201 (57.8)
What is the best anti-diabetic agent to be combined with SGLT2 inhibitors?	Metformin	206 (59.2)
SGLT2 inhibitors are contraindicated in?	Patients with renal failure	221 (63.5)
What is the superiority of SGLT2 inhibitors over sulfonylurea?	Have better protective effect in patients with diabetes and hypertension	222 (63.8)
Dosage form?	Tablets	322 (92.5)

### Community pharmacists’ views and dispensing practice toward SGLT2 inhibitors

More than half of the respondent pharmacists thought that SGLT2 inhibitors have a better effect when they are prescribed to a particular group of patients (n=195, 56%). However, more than 20% of them thought that they are slightly better than the available anti-diabetic medications (n=76, 21.8%). Moreover, 49.1% of the pharmacists reported that they received one to five SGLT2 inhibitor prescriptions per month, and only 8.3% reported that they received more than ten prescriptions per month. Around half of the pharmacists said that the patient’s feedback about SGLT2 inhibitors was very good (n=168, 48.3%). However, very few pharmacists said that the patients had a bad impression of these medications (n=5, 1.4%). A total of 54.9% of the pharmacists revealed that the most common feedback from the patients about these agents is their high price compared to the other anti-diabetic medications. Furthermore, half of the pharmacists thought that their knowledge about SGLT2 inhibitors was good (n=174, 50%), however, 21.6% thought they had a weak knowledge. The vast majority of the pharmacists had not attended a training course about SGLT2 inhibitors before (n=269, 77.3%). In addition, the vast majority of respondents demonstrated that they were in high (n=150,43.1%) to moderate (n=161, 46.3%) need of training courses about SGLT2 inhibitors.

### Factors influencing pharmacists’ knowledge about SGLT2

An independent sample t-test was performed to examine the differences in knowledge levels based on the demographics of the pharmacists and other factors with two categories that might affect their knowledge level which are shown in Table 3. The test demonstrated that age and gender of the pharmacists had no significant effect on their knowledge level. In addition, there was no significant difference in pharmacist’s knowledge based on pharmacy location, pharmacist’s working time (either morning or afternoon or night) and their bachelor’s degree major (either pharmacy or pharmacy Doctor). The t-test indicated that holding a postgraduate degree did not affect the pharmacist’s knowledge. Interestingly, the test showed that the pharmacists who had attended training courses on diabetes before had a higher knowledge score compared to those who had not attended training courses (p=0.02).

**Table 3.** Differences in knowledge based on demographics with two categories.

Demographics	Categories	Mean (SD)	t	DF	P value
Age	Up to 29	6.68 (2.12)	0.79	346	0.43
	30 or more	6.48 (2.38)			
Gender	Female	6.76 (2.27)	1.53	346	0.127
	Male	6.4 (2.12)			
Pharmacy location	City	6.58 (2.24)	0.95	346	0.33
	Countryside	7.11 (1.65)			
Working time	Morning	6.79 (2.18)	1.55	346	0.12
	Afternoon or night	6.42 (2.23)			
Postgraduate degree	Higher diploma or master	6.20 (2.44)	-1.31	346	0.18
	Have no postgraduate degree	6.67 (2.18)			
Training courses in diabetes	Yes	6.78 (2.16)	-2.31	346	0.02
	No	6.16 (2.31)			
Bachelor’s degree major	Pharmacy	6.55 (2.22)	-1.48	346	0.13
	Pharm D	7.09 (2.10)			

A one-way ANOVA test was used to examine the differences in knowledge level in relation to variables which had more than two categories, and results are shown in Table 4. The results showed that the years of experience of the pharmacists has no significant effect on their knowledge about SGLT2 inhibitors. Similarly, the number of prescriptions received by the pharmacist per month, the feedback of the patients who were using SGLT2 inhibitors, and the source of information that the pharmacists used to gain knowledge about SGLT2 inhibitors, had no significant effect on pharmacist’s knowledge. However, pharmacists who thought that SGLT2 inhibitors had no superiority over the other available anti-diabetic medications had lower knowledge scores compared to those who thought the opposite (Scheffe posthoc, p=0.018). In addition, results showed that pharmacists who thought that SGLT2 inhibitors have a better effect when they are administered to a particular group of patients had a higher knowledge score (Scheffe posthoc, 0.0001). Moreover, pharmacists who thought that the best advice they could give for patients who use SGLT2 inhibitors is to keep genital area clean to avoid infection had a higher knowledge score compared to those who thought the best advice was monitoring blood glucose to avoid hypoglycaemia events or monitor body weight to avoid weight gain, or compared to those who had no specific advice to give to the patients (Scheffe posthoc, p=0.0001). Surprisingly, pharmacists who thought they did not face any obstacles while prescribing SGLT2 inhibitors had significantly lower knowledge scores compared to those who thought that the price of these medications is the main obstacle they encounter (Scheffe posthoc, p=0.05). Expectedly, pharmacists who considered their knowledge is excellent had higher knowledge scores compared to those who considered their knowledge was weak (Scheffe posthoc, p=0.04). Furthermore, pharmacists who assessed their need for training courses about SGLT2 inhibitors as moderate had higher knowledge scores compared to those who thought their need for this kind of courses was high or thought there was no need for these courses (Scheffe posthoc, p=0.003, 0.004; respectively).

**Table 4.** Differences in knowledge based on demographics with more than 2 categories.

Demographics/Dispensing practice	Categories	Mean (SD)	f	DF	P value
Years of experience	0-4 years	6.64 (2.13)	0.07	345	0.93
	5-9 years	6.54 (2.36)			
	10 years or more	6.61 (2.28)			
What is your impression about SGLT2 inhibitors?	Have no superiority over the available medications	5.09 (2.09)	11.98	344	0.0001
	Slightly better than the available medications	6.38 (1.72)			
	Give better effect when they are administered to a particular group of patients	7.12 (2.21)			
	They are expensive medications without any added benefits to the patients	6.14 (2.37)			
How many prescriptions per month do you receive for this group?	One prescription or less	6.29 (2.15)	3.03	344	0.29
	1-5 prescriptions	6.67 (2.19)			
	5-10 prescriptions	6.43 (2.59)			
	More than 10 prescriptions	7.65 (1.56)			
How do you see the feedback of patients who are using these drugs?	Good	6.33 (2.02)	2.27	344	0.080
	Very good	6.89 (2.39)			
	Excellent	6.27 (1.99)			
	Bad impression	7.60 (1.67)			
What are the obstacles you face when you are dispensing these drugs?	Patients lack knowledge	6.25 (1.72)	2.56	344	0.05
	Lack of my knowledge	6.40 (2.00)			
	Patients comments about the price	6.90 (2.35)			
	I have no obstacles	6.09 (2.17)			
What do you think the evaluation of your knowledge about these drugs?	Excellent	7.27 (2.53)	2.64	344	0.04
	Very good	6.71 (2.18)			
	Good	6.70 (2.29)			
	Weak	6.05 (1.84)			
How do you assess your need to attend training courses about SGLT2 inhibitors?	High need	6.26 (2.20)	8.91	345	0.0001
	Moderate need	7.12 (2.14)			
	No need currently because it is rarely used	5.81 (2.11)			
What is the source of information which you use to get your knowledge about SGLT2 inhibitors?	Scientific journals	6.22 (2.28)	2.02	344	0.11
	Websites	6.91 (2.23)			
	Pharmaceutical companies	6.61 (2.20)			
	Other sources	6.32 (2.08)			

## Discussion

The present study has assessed pharmacists’ knowledge level, views, and practice about a new anti-diabetic group of medications, SGLT2 inhibitors, in Jordan. In addition, the study explored the factors which could influence their knowledge level. No surveys have been conducted to assess pharmacists’ knowledge and views toward SGLT2 inhibitors in Middle East countries, and to our knowledge this is the first questionnaire survey that was conducted in Jordan.

This study revealed a fair knowledge and understanding about SGLT2 inhibitors pharmacotherapy among the study sample. Our results indicate that there was no significant impact of gender and age of the pharmacists on their knowledge level. This finding is consistent with a previous study which showed that the knowledge of pharmacists was not affected by their age and gender. ^19^^,^^20^ Notably, the findings showed that there was no significant difference in knowledge level between respondents based on their pharmacy location (cities or countryside) or their postgraduate degree. These results are inconsistent with previous reports which showed that pharmacists’ knowledge in rural areas was poor compared to pharmacists’ knowledge in urban areas. ^21^^,^^22^ This finding could be partly explained by the fact that the majority of the participants in our study were working in urban areas, and they did not hold postgraduate degrees. Additionally, SGLT2 inhibitors were introduced recently to the market in Jordan, which was many years after their graduation (more than 45% of respondents had more than five years of experience). Moreover, possibly due to high price of these agents, ^23^ they have been introduced selectively to certain pharmacies, so many pharmacists were unexposed to these medications. Consistent with the literature, this research found that community pharmacists who attended training courses on diabetes had a higher knowledge about SGLT2 inhibitors. ^24^^,^^25^ In fact, previous studies have shown that continuous training for pharmacists and engaging them in diabetes self-management training programmes is essential to improve their skills and role in assisting the patients they serve. ^24^^,^^25^ However, the majority of the participants indicated that they were in high or moderate need for training courses about SGLT2 inhibitors. ^8^ This response was reflected in their average score of knowledge about SGLT2 inhibitors. Moreover, the present study found non-significant differences in the knowledge score of the pharmacists regarding SGLT2 inhibitors based on their demographics data and in association with their different factors. The low score of knowledge about SGLT2 inhibitors can be explained by various factors including the insufficient courses about diabetes in pharmacy schools, ^26^^,^^27^ and the inadequate continuous pharmaceutical education for the community pharmacists after graduation, ^28^^,^^29^ particularly, a training program on the newly registered pharmaceutical products such as SGLT2 inhibitors. ^30^ Taken together, the lack of knowledge about SGLT2 inhibitors might affect community pharmacists’ practice, which may lead to negative impact on patients’ outcomes. ^31^^,^^32^

Furthermore, this study highlights the views and dispensing practice of community pharmacists in Jordan towards SGLT2 inhibitors. More than half of the respondent pharmacists supported that SGLT2 inhibitors have a better effect when they are prescribed to a particular group of patients. This view concords with the fact that SGLT2 inhibitors are useful for a particular group of patients such as obese or hypertensive patients with diabetes, or patients who are at higher risk of hypoglycaemia. ^33^^,^^34^ Importantly, SGLT2 inhibitors provide greater HbA1c reduction when compared with sulphonylureas or other oral anti-diabetic agents. ^35^^,^^36^ Our results demonstrated that most pharmacists think that these agents have no superiority over the other available anti-diabetic medications; however, many studies show the superiority of SGLT2 inhibitors and different classes of new anti-diabetic agents in reducing the HbA1c, ^37^^–^^39^ which could be explained by their lack of knowledge about these anti-diabetic agents. Around half of the patients provided positive feedback about these agents. This finding is consistent with that of previous studies which indicated that SGLT2 inhibitors improved clinical treatment satisfaction in people with diabetes. ^40^^,^^41^ However, positive and negative feedback from patients about these agents have been reported. ^42^ Note that the last American Diabetes Association (ADA) guideline in 2018 has recommended to combine SGLT2 inhibitors with metformin for their benefits in decreasing the risk of cardiovascular events. ^43^ Therefore, with this recommendation, more physicians are expected to prescribe these medications, ^44^ which supports the need for increasing pharmacists’ knowledge about SGLT2 inhibitors.

Furthermore, only about one third of the pharmacists in this study could give the proper advice for patients with diabetes who are using SGLT2 inhibitors, which is to keep genital area clean to avoid infection. ^35^ This is an important advice that should be given to the patients who use SGLT2 inhibitors, because these agents increase the glucose excretion in the urinary tract, which pre-dispose patients to genital tract infections. These infections are usually fungal in nature, and can present as vulvitis in women, and balanaposthitis or balanitis in men. ^45^^–^^47^

## Conclusions

The findings of this study demonstrated that pharmacist’s knowledge about SGLT2 inhibitors is moderate, which may negatively affect the outcomes of this medication on patients. In addition, it shed light on the importance of continuous education for the community pharmacists on these new anti-diabetic agents, because pharmacists have essential roles in the healthcare system due to their accessibility to patients. The findings of this study encourage to conduct further research on the awareness of pharmacists of these medications in all healthcare settings.

## Data availability

### Underlying data

Open Science Framework. Knowledge and Practice of Community Pharmacists towards SGLT2 Inhibitors, https://doi.org/10.17605/OSF.IO/W928T.

This project contains the following underlying data:

‐ SGLT2 inhibitors data.sav

Data are available under the terms of the Creative Commons Attribution 4.0 International license (CC-BY 4.0).

## References

[1] Blair M : Diabetes Mellitus Review. *Urol. Nurs.* 2016;36:27–36. 10.7257/1053-816X.2016.36.1.27 27093761

[2] Silva JA Da : Diagnosis of diabetes mellitus and living with a chronic condition: Participatory study. *BMC Public Health.* 2018;18:699. 10.1186/s12889-018-5637-9 29871637 PMC5989432

[3] Chatterjee S Khunti K Davies MJ : Type 2 diabetes. *Lancet.* 2017;389:2239–2251. 10.1016/S0140-6736(17)30058-2 28190580

[4] *WHO|About diabetes.* WHO;2014.

[5] Inzucchi SE : Management of hyperglycemia in type 2 diabetes, 2015: a patient-centered approach: update to a position statement of the American Diabetes Association and the European Association for the Study of Diabetes. *Diabetes Care.* 2015;38:140–149. 10.2337/dc14-2441 25538310

[6] Scheen AJ : Pharmacodynamics, efficacy and safety of sodium-glucose co-transporter type 2 (SGLT2) inhibitors for the treatment of type 2 diabetes mellitus. *Drugs.* 2015;75:33–59. 10.1007/s40265-014-0337-y 25488697

[7] Watts NB : Effects of canagliflozin on fracture risk in patients with type 2 diabetes mellitus. *J. Clin. Endocrinol. Metab.* 2016;101:157–166. 10.1210/jc.2015-3167 26580237 PMC4701850

[8] Moses RG Colagiuri S Pollock C : SGLT2 inhibitors: New medicines for addressing unmet therapeutic needs in type 2 diabetes. *Australas. Med. J.* 2014;7:405–415. 10.4066/AMJ.2014.2181 25379062 PMC4221776

[9] Desouza CV Gupta N Patel A : Cardiometabolic Effects of a New Class of Antidiabetic Agents. *Clin. Ther.* 2015;37:1178–1194. 10.1016/j.clinthera.2015.02.016 25754876

[10] Zinman B : Empagliflozin, cardiovascular outcomes, and mortality in type 2 diabetes. *N. Engl. J. Med.* 2015;373:2117–2128. 10.1056/NEJMoa1504720 26378978

[11] Handelsman Y : American association of clinical endocrinologists and American college of endocrinology - Clinical practice guidelines for developing a diabetes mellitus comprehensive care plan - 2015. *Endocr. Pract.* 2015;21 Suppl 1:1–87. 10.4158/EP15672.GL 25869408 PMC4959114

[12] IDF Diabetes Atlas|Tenth Edition. Reference Source

[13] Ajlouni K : Time trends in diabetes mellitus in Jordan between 1994 and 2017. *Diabet. Med.* 2019;36:1176–1182. 10.1111/dme.13894 30614070

[14] Kovacich N Chavez B : Ertugliflozin (Steglatro): A New Option for SGLT2 Inhibition. *Pharm. Ther.* 2018;43:736. PMC6281144 30559584

[15] Sharaf SE : Knowledge, attitude, practice, and pharmaceutical outcomes of type 2 diabetes mellitus selfmanagement among patients in Makkah Region, Saudi Arabia. *Pharm. Pharmacol. Int. J.* 2021;9:94–101. 10.15406/ppij.2021.09.00333

[16] Hassali MA Nazir SUR Saleem F : Literature review: Pharmacists’ interventions to improve control and management in type 2 diabetes mellitus. *Altern. Ther. Health Med.* 2015;21:28–35. 25599430

[17] Singh RF : Evaluation of a short, interactive diabetes self-management program by pharmacists for type 2 diabetes. *BMC. Res. Notes.* 2018;11:828. 10.1186/s13104-018-3952-y 30477584 PMC6260875

[18] Jamshed SQ Siddiqui MJ Rana B : Evaluation of the Involvement of Pharmacists in Diabetes Self-Care: A Review From the Economic Perspective. *Front. Public Heal.* 2018;6. 10.3389/fpubh.2018.00244 30234088 PMC6129588

[19] Abahussain NA Abahussain EA Al-Oumi FM : Pharmacists’ attitudes and awareness towards the use and safety of herbs in Kuwait. *Pharm. Pract. (Granada).* 2007;5:125–129. 10.4321/S1886-36552007000300005 25214928 PMC4154746

[20] Della Polla G : Knowledge, attitudes, and practices towards infectious diseases related to travel of community pharmacists in italy. *Int. J. Environ. Res. Public Health.* 2020;17. 10.3390/ijerph17062147 32213832 PMC7143491

[21] Ansari M : Evaluation of community pharmacies regarding dispensing practices of antibiotics in two districts of central Nepal. *PLoS One.* 2017;12:e0183907. 10.1371/journal.pone.0183907 28949990 PMC5614431

[22] Bagher AM : Knowledge, perception, and confidence of hospital pharmacists toward pharmacogenetics in Jeddah, Kingdom of Saudi Arabia. *Saudi Pharm. J.* 2021;29:53–58. 10.1016/j.jsps.2020.12.006 33603539 PMC7873749

[23] Luo J Feldman R Rothenberger SD : Coverage, Formulary Restrictions, and Out-of-Pocket Costs for Sodium-Glucose Cotransporter 2 Inhibitors and Glucagon-Like Peptide 1 Receptor Agonists in the Medicare Part D Program. *JAMA Netw. Open.* 2020;3:e2020969. 10.1001/jamanetworkopen.2020.20969 33057641 PMC7563069

[24] Mahmoudi L Shafiekhani M Dehghanpour H : Community pharmacists’ knowledge, attitude, and practice of irritable bowel syndrome (Ibs): The impact of training courses. *Adv. Med. Educ. Pract.* 2019;10:427–436. 10.2147/AMEP.S201904 31417329 PMC6592030

[25] Thakur T Galt KA Siracuse MV : National survey of diabetes self-management program coordinators views about pharmacists’ roles in diabetes education. *J. Am. Pharm. Assoc.* 2020;60:336–343.e1. 10.1016/j.japh.2019.10.009 31859219

[26] Westberg SM Bumgardner MA Brown MC : Impact of an elective diabetes course on student pharmacists’ skills and attitudes. *Am. J. Pharm. Educ.* 2010;74:49. 10.5688/aj740349 20498742 PMC2865415

[27] Shrader S Kavanagh K Thompson A : A Diabetes Self-Management Education Class Taught by Pharmacy Students. *Am. J. Pharm. Educ.* 2012;76:13. 10.5688/ajpe76113 22412212 PMC3298395

[28] Ayadurai S Sunderland B Tee LBG : A training program incorporating a diabetes tool to facilitate delivery of quality diabetes care by community pharmacists in Malaysia and Australia. *Pharm. Pract. (Granada).* 2019;17:1457. 10.18549/PharmPract.2019.2.1457 31275501 PMC6594426

[29] MacCallum L : Pharmacists report lack of reinforcement and the work environment as the biggest barriers to routine monitoring and follow-up for people with diabetes: A survey of community pharmacists. *Res. Social Adm. Pharm.* 2021;17:332–343. 10.1016/j.sapharm.2020.04.004 32327399

[30] Lam D Shaikh A : Real-Life Prescribing of SGLT2 Inhibitors: How to Handle the Other Medications, Including Glucose-Lowering Drugs and Diuretics. *Kidney360.* 2021;2:742–746. 10.34067/KID.0000412021 35373039 PMC8791327

[31] Watson LL Bluml BM : Integrating pharmacists into diverse diabetes care teams: Implementation tactics from Project IMPACT: Diabetes. *J. Am. Pharm. Assoc.* 2014;54:538–541. 10.1331/JAPhA.2014.14063 25216884

[32] Cranor CW Christensen DB : The Asheville Project: Short-term outcomes of a community pharmacy diabetes care program. *J. Am. Pharm. Assoc.* 2003;43:160–172. 10.1331/108658003321480704 12688433

[33] Masson W Lavalle-Cobo A Nogueira JP : Effect of SGLT2-Inhibitors on Epicardial Adipose Tissue: A Meta-Analysis. *Cells.* 2021;10. 10.3390/cells10082150 34440918 PMC8391573

[34] Sørensen AMS Christensen MB : Cardiovascular effects of antidiabetic drugs. *Drugs Today (Barc).* 2018;54:547. 10.1358/dot.2018.54.9.2872500 30303495

[35] Opie LH : Sodium glucose co-transporter 2 (SGLT2) inhibitors: new among antidiabetic drugs. *Cardiovasc. Drugs Ther.* 2014;28:331–334. 10.1007/s10557-014-6522-0 24825435

[36] Sipos Á : Dual-Target Compounds against Type 2 Diabetes Mellitus: Proof of Concept for Sodium Dependent Glucose Transporter (SGLT) and Glycogen Phosphorylase (GP) Inhibitors. *Pharmaceuticals (Basel).* 2021;14. 10.3390/ph14040364 33920838 PMC8071193

[37] Jia S : Incretin mimetics and sodium-glucose co-transporter 2 inhibitors as monotherapy or add-on to metformin for treatment of type 2 diabetes: a systematic review and network meta-analysis. *Acta Diabetol.* 2021;58:5–18. 10.1007/s00592-020-01542-4 32514989

[38] Wang Z : Efficacy and safety of sodium-glucose cotransporter-2 inhibitors versus dipeptidyl peptidase-4 inhibitors as monotherapy or add-on to metformin in patients with type 2 diabetes mellitus: A systematic review and meta-analysis. *Diabetes. Obes. Metab.* 2018;20:113–120. 10.1111/dom.13047 28656707

[39] Sundström J : A registry-based randomised trial comparing an SGLT2 inhibitor and metformin as standard treatment of early stage type 2 diabetes (SMARTEST): Rationale, design and protocol. *J. Diabetes Complicat.* 2021;35:107996. 10.1016/j.jdiacomp.2021.107996 34389234

[40] Ishibashi R : Assessing Patient Satisfaction Following Sodium Glucose Co-Transporter 2 Inhibitor Treatment for Type 1 Diabetes Mellitus: A Prospective Study in Japan. *Diabetes Ther.* 2021;12:453–460. 10.1007/s13300-020-00971-2 33237553 PMC7843726

[41] Suzuki D : Sodium-glucose cotransporter 2 inhibitors improved time-in-range without increasing hypoglycemia in Japanese patients with type 1 diabetes: A retrospective, single-center, pilot study. *J. Diabetes Investig.* 2020;11:1230–1237. 10.1111/jdi.13240 32100964 PMC7477508

[42] Nakajima H : Dapagliflozin improves treatment satisfaction in overweight patients with type 2 diabetes mellitus: a patient reported outcome study (PRO study). *Diabetol. Metab. Syndr.* 2018;10:11. 10.1186/s13098-018-0313-x 29507611 PMC5831584

[43] Lopaschuk GD Verma S : Mechanisms of Cardiovascular Benefits of Sodium Glucose Co-Transporter 2 (SGLT2) Inhibitors: A State-of-the-Art Review. *JACC. Basic Transl. Sci.* 2020;5:632–644. 10.1016/j.jacbts.2020.02.004 32613148 PMC7315190

[44] Alzarah HA : Evaluation of pharmacists’ knowledge of sodium-glucose cotransporter 2 inhibitors in the eastern province, Saudi Arabia. *Int. J. Med. Dev. Ctries.* 1046–1050. 10.24911/IJMDC.51-1549226975

[45] Brown E Rajeev SP Cuthbertson DJ : A review of the mechanism of action, metabolic profile and haemodynamic effects of sodium-glucose co-transporter-2 inhibitors. *Diabetes. Obes. Metab.* 2019;21 Suppl 2:9–18. 10.1111/dom.13650 31081592

[46] Johnsson KM : Vulvovaginitis and balanitis in patients with diabetes treated with dapagliflozin. *J. Diabetes Complicat.* 2013;27:479–484. 10.1016/j.jdiacomp.2013.04.012 23806570

[47] McGovern AP : Risk factors for genital infections in people initiating SGLT2 inhibitors and their impact on discontinuation. *BMJ Open Diabetes Res. Care.* 2020;8:e001238. 10.1136/bmjdrc-2020-001238 32448787 PMC7252998

