# Supplementary material for: Knowledge and practice of community pharmacists towards SGLT2 inhibitors
Source: F1000Res. 2022 Jun 30;11:659. Originally published 2022 Jun 15. [Version 2] doi: 10.12688/f1000research.122170.2 (PMC9237554; doi:10.12688/f1000research.122170.2)
Supplement: Peer review history

# Peer review: Reviewer response for version 2

**DOI:** 10.5256/f1000research.135641.r142795

**Published:** 2022-07-05

**Authors:** Hala Jehad Mahdi Al-Obaidi

**Affiliations:** 1 College of Pharmacy and Health Sciences, Ajman University, Ajman, United Arab Emirates

Approved. I have added a few more comments to the revised attached article ( here). Please find them on Page 4, 6, and 9 these are my last comments on this article.

Is the work clearly and accurately presented and does it cite the current literature?

Yes

If applicable, is the statistical analysis and its interpretation appropriate?

Partly

Are all the source data underlying the results available to ensure full reproducibility?

Yes

Is the study design appropriate and is the work technically sound?

Yes

Are the conclusions drawn adequately supported by the results?

Yes

Are sufficient details of methods and analysis provided to allow replication by others?

Partly

Reviewer Expertise:

Diabetic patient’s knowledge, attitude and Practice using questionnaire in a cross sectional method.

I confirm that I have read this submission and believe that I have an appropriate level of expertise to confirm that it is of an acceptable scientific standard.

# Peer review: Reviewer response for version 1

**DOI:** 10.5256/f1000research.134124.r140909

**Published:** 2022-06-27

**Authors:** Belal Azab

**Affiliations:** 1 Department of Pathology and Cell Biology, Columbia University Irving Medical Center, New York, NY, USA

A well-written manuscript that assessed the knowledge practice of community pharmacists about SGLT2 inhibitors in Jordan. This manuscript gets to a great conclusion that is well supported by extensively analyzed data. I have some minor comments below that won’t affect the robustness of the manuscript, but they are just opportunities for improvement.

- In paragraph 3 in the introduction, add a reference after ‘’people with diabetes’’ line number 9.
- At the end of the introduction, it would be better to elaborate more on the impact of this study which explains the aims of this study.
- The section entitled ‘’Development of the survey questionnaire’’ in the methods should be amended to ‘’Development of study instrument’’
- I think the first paragraph in the discussion is a repetition, so, should be deleted.

Overall, this is an interesting study and the authors have collected and analyzed a good dataset using appropriate methodology. The paper is generally well written and structured, and I recommend it for indexing.

Is the work clearly and accurately presented and does it cite the current literature?

Yes

If applicable, is the statistical analysis and its interpretation appropriate?

Yes

Are all the source data underlying the results available to ensure full reproducibility?

Yes

Is the study design appropriate and is the work technically sound?

Yes

Are the conclusions drawn adequately supported by the results?

Yes

Are sufficient details of methods and analysis provided to allow replication by others?

Yes

Reviewer Expertise:

Molecualar biology, pharmacogenetics, human genetics

I confirm that I have read this submission and believe that I have an appropriate level of expertise to confirm that it is of an acceptable scientific standard.

# Response

**Published:** 2022-06-28

**Authors:** Abdelrahim qudhah

The authors would like to thank you for your positive valuable feedback on the manuscript which will improve the quality of it.

 C1: In paragraph 3 in the introduction, add a reference after ‘’people with diabetes’’ line number 9.

***R1: A reference is now added.***

 C2: At the end of the introduction, it would be better to elaborate more on the impact of this study which explains the aims of this study.

***R2: The impact of this study is added now.***

 C3:The section entitled ‘’Development of the survey questionnaire’’ in the methods should be amended to ‘’Development of study instrument’’

***R3: The title of this section is amended as recommended.***

 C4: I think the first paragraph in the discussion is a repetition, so, should be deleted.

***R4: This paragraph is now deleted.***

# Peer review: Reviewer response for version 1

**DOI:** 10.5256/f1000research.134124.r140911

**Published:** 2022-06-27

**Authors:** Hala Jehad Mahdi Al-Obaidi

**Affiliations:** 1 College of Pharmacy and Health Sciences, Ajman University, Ajman, United Arab Emirates

The study is very well designed and well written. I enjoyed reading it. Decision made: Approved with only minor changes are required as follows:

**Method:**

 In the data analysis section, it is stated that the " *Data were analysed by using SPSS software after extracting it from Google forms*", this means that the questionnaire was computer-based, i.e. iPads/PCs were used, however, in the design and data collection section, you’ve mentioned that the questionnaire was distributed face-to-face with participants at their workplace, which made me think that the questionnaire was a paper-based survey. Please clarify this point.

**Development of the survey questionnaire:**

 It is mentioned that the questionnaire was composed of 28 questions which were divided into three sections: The first section consisted of eight questions; the second section was composed of 12 questions and the third section was composed of nine questions. The total number of 8, 12, and 9 is 29, not 28. Please check the accurate number of the questions under each section accordingly.

**Data Analysis:**

 It is mentioned (in data analysis section) that ‘ *Demographics numerical variables were described using **mean and standard deviation***’. This was used in Table 3 and Table 4 (Knowledge difference in demographics with two categories) i.e. the mean (±SD). While in other Tables, i.e. Table 1 (demographic details) and Table 2 (Community pharmacists’ knowledge about SGLT2 inhibitors) the total number and percentages (no.; %) were used not Mean and SD. You can specify those separately. Also, it is recommended to mention the name of the statistical test used either under each Table or in the data analysis section.

**Knowledge scale validity and score:**

 In this section, full details of the scores are needed, i.e. not only from 1 (not all) to 4 (strongly agreed). It is well explained how the knowledge scale is validated but it is not clear how the SGLT2 inhibitor's knowledge was calculated to 6.61 (SD=2.22, range 1-12). You may explain here more about it and state the range of the high, moderate, or low/poor knowledge scores to compare your findings with the knowledge scale average. And explain how the pharmacist’s knowledge is considered a low or moderate level.

**Discussion:**

 Across the discussion part, it is recommended to add the *P-*value for each significant/not significant finding. Also, in the discussion section, it is stated that **“ *Around half of the patients provided positive feedback about these agents*”**. It is better to write the exact percentage and reference to support this statement.

**Conclusion:**

 In the conclusion, it is stated that ****the findings of this study demonstrated that pharmacists’ knowledge about SGLT2 inhibitors is Moderate. It is recommended to add the score for this evaluation.

Is the work clearly and accurately presented and does it cite the current literature?

Yes

If applicable, is the statistical analysis and its interpretation appropriate?

Partly

Are all the source data underlying the results available to ensure full reproducibility?

Yes

Is the study design appropriate and is the work technically sound?

Yes

Are the conclusions drawn adequately supported by the results?

Yes

Are sufficient details of methods and analysis provided to allow replication by others?

Partly

Reviewer Expertise:

Diabetic patient’s knowledge, attitude and Practice using questionnaire in a cross sectional method.

I confirm that I have read this submission and believe that I have an appropriate level of expertise to confirm that it is of an acceptable scientific standard, however I have significant reservations, as outlined above.

# Response

**Published:** 2022-06-28

**Authors:** Abdelrahim qudhah

The authors would like to thank you for your positive valuable feedback on the manuscript which will improve the quality of it.

 The study is very well designed and well written. I enjoyed reading it. Decision made: Approved with only minor changes are required as follows:

**Method:**

 C1: In the data analysis section, it is stated that the " *Data were analysed by using SPSS software after extracting it from Google forms*", this means that the questionnaire was computer-based, i.e. iPads/PCs were used, however, in the design and data collection section, you’ve mentioned that the questionnaire was distributed face-to-face with participants at their workplace, which made me think that the questionnaire was a paper-based survey. Please clarify this point.

***R1: Thank you for your comment. The questionnaire was computer-based, however, it was distributed to the participants face-to-face, not over social media platforms.***

**C2: Development of the survey questionnaire:**

 It is mentioned that the questionnaire was composed of 28 questions which were divided into three sections: The first section consisted of eight questions; the second section was composed of 12 questions and the third section was composed of nine questions. The total number of 8, 12, and 9 is 29, not 28. Please check the accurate number of the questions under each section accordingly.

***R2: Thank you for your comment. The third section was composed of eight questions. It is now corrected.***

**C3: Data Analysis:**

 It is mentioned (in data analysis section) that ‘ *Demographics numerical variables were described using **mean and standard deviation***’. This was used in Table 3 and Table 4 (Knowledge difference in demographics with two categories) i.e. the mean (±SD). While in other Tables, i.e. Table 1 (demographic details) and Table 2 (Community pharmacists’ knowledge about SGLT2 inhibitors) the total number and percentages (no.; %) were used not Mean and SD. You can specify those separately. Also, it is recommended to mention the name of the statistical test used either under each Table or in the data analysis section.

***R3: Thank you for your comment. The ‘’mean and standard deviation is amended to ‘’frequencies’’. The test used is now added beside the title of each table.***

**C4: Knowledge scale validity and score:**

 In this section, full details of the scores are needed, i.e. not only from 1 (not all) to 4 (strongly agreed). It is well explained how the knowledge scale is validated but it is not clear how the SGLT2 inhibitor's knowledge was calculated to 6.61 (SD=2.22, range 1-12). You may explain here more about it and state the range of the high, moderate, or low/poor knowledge scores to compare your findings with the knowledge scale average. And explain how the pharmacist’s knowledge is considered a low or moderate level.

***R4: Thank you for your comment. This section is now amended. Full details of the scores were added. The knowledge score was calculated as an average for the adequate answers of the participants. Knowledge score was classified as poor (1-4), moderate (4-8), and high (8-12).***

**C5: Discussion:**

 Across the discussion part, it is recommended to add the *P-*value for each significant/not significant finding. Also, in the discussion section, it is stated that **“ *Around half of the patients provided positive feedback about these agents*”**. It is better to write the exact percentage and reference to support this statement.

***R5: Thank you for your comment. P value and percentage are now added.***

**C6: Conclusion:**

 In the conclusion, it is stated that ****the findings of this study demonstrated that pharmacists’ knowledge about SGLT2 inhibitors is Moderate. It is recommended to add the score for this evaluation.

***R6: The score is added now.***

# Peer review: Reviewer response for version 1

**DOI:** 10.5256/f1000research.134124.r140908

**Published:** 2022-06-23

**Authors:** Ayman G. Mustafa

**Affiliations:** 1 Department of Basic Medical Sciences, College of Medicine, QU Health, Qatar University, Doha, Qatar

This paper discusses community pharmacists’ knowledge and practices toward a group of a new anti-diabetic group, SGLT2 inhibitors, in Jordan which is an important topic that gives new insight into this group of medications.

 The paper is well-written and of high quality, however, I have some minor comments that would improve the paper:

- In the abstract, Add the expected impact of this study, for example; Improving pharmacists’ knowledge about this group of medications could improve the treatment outcome of people with diabetes.
- In the introduction, in the first paragraph, line 5, insert reference after ‘’metformin monotherapy’’
- Also in the second paragraph of the introduction, line 5, insert reference after ‘’glucose level’’
- In methods, the section entitled ‘Knowledge scale validity and score’’ is best be moved up before the statistical analysis section.

The results of this study are quite novel which could help the pharmacists to improve their knowledge about this group of medications that will improve the quality of life for people with diabetes. I think the study is of good value and I recommend it for indexing.

Is the work clearly and accurately presented and does it cite the current literature?

Yes

If applicable, is the statistical analysis and its interpretation appropriate?

Yes

Are all the source data underlying the results available to ensure full reproducibility?

Yes

Is the study design appropriate and is the work technically sound?

Yes

Are the conclusions drawn adequately supported by the results?

Yes

Are sufficient details of methods and analysis provided to allow replication by others?

Yes

Reviewer Expertise:

Free radicals in biology and medicine

I confirm that I have read this submission and believe that I have an appropriate level of expertise to confirm that it is of an acceptable scientific standard.

# Response

**Published:** 2022-06-28

**Authors:** Abdelrahim qudhah

The authors would like to thank you for your valuable feedback on the manuscript which will improve the quality of it.

 C1: In the abstract, Add the expected impact of this study, for example; Improving pharmacists’ knowledge about this group of medications could improve the treatment outcome of people with diabetes.

***R1: The expected impact of this study was added to the abstract.***

******

 C2: In the introduction, in the first paragraph, line 5, insert reference after ‘’metformin monotherapy’’

***R2: A reference is now added.***

 C3: Also in the second paragraph of the introduction, line 5, insert reference after ‘’glucose level’’

***R3: A reference is now added.***

 C4: In methods, the section entitled ‘Knowledge scale validity and score’’ is best be moved up before the statistical analysis section.

***R4: The title of this section is amended as recommended.***
